# Design of a high-efficiency gilbert mixer with current source assistance and current bleeding for HEV and wireless sensor network applications

**DOI:** 10.1038/s41598-026-56889-5

**Published:** 2026-06-11

**Authors:** Shasanka Sekhar Rout, Abhijit Mishra, Asisa Kumar Panigrahy, Vakkalakula Bharath Sreenivasulu

**Affiliations:** 1 Department of ECE, GLA University, Mathura, Uttar Pradesh 281406 India; 2 School of Electronics Engineering (SENSE), Vellore Institute of Technology, Chennai, Tamil Nadu 600127 India; 3 Department of ECE, ICFAI Foundation for Higher Education Hyderabad, Hyderabad, Telangana 501203 India; 4 Manipal Institute of Technology Bengaluru, Manipal Academy of Higher Education, Manipal, India

**Keywords:** Current source helpers, Current bleeding, HEV, Linearity, IIP3, V2X, Energy science and technology, Engineering

## Abstract

The effective creation of better, faster, and more efficient electronic circuits particularly in the radio frequency (RF) segment, is more important than ever for the entire world. A prevalent issue in wireless communication systems, such as those used for charging or Vehicle-to-Everything (V2X) communication, interference and signal reception problems are related to the receiver front end of electric vehicles (EV) applications. Modern receiver technologies are one way to address this issue, as they reduce interference and enhance signal reception. To address the above requirements, this paper introduces a down conversion Gilbert mixer with the improvement in conversion gain, power efficiency, linearity and noise performance. Here, the proposed mixer design uses current source helpers and current bleeding technique to enhance the performance of the conventional Gilbert mixer. The proposed mixer is simulated in Cadence Tool with an intermediate frequency (IF) of 100 MHz, which provides a conversion gain of 12 dB with the third order input intercept point (IIP3) of 4.5 dBm and a noise figure (NF) of about 9.86 dB. Also, the design functions at a supply voltage of 1.2 V, with a power consumption of only 0.96 mW. So, this mixer design may be the correct choice as a core component in the receiver front end in applications like HEVs and sensor networks, especially where wireless communication, signal conversion, and sensing are involved.

## Introduction

**Fig. 1:**
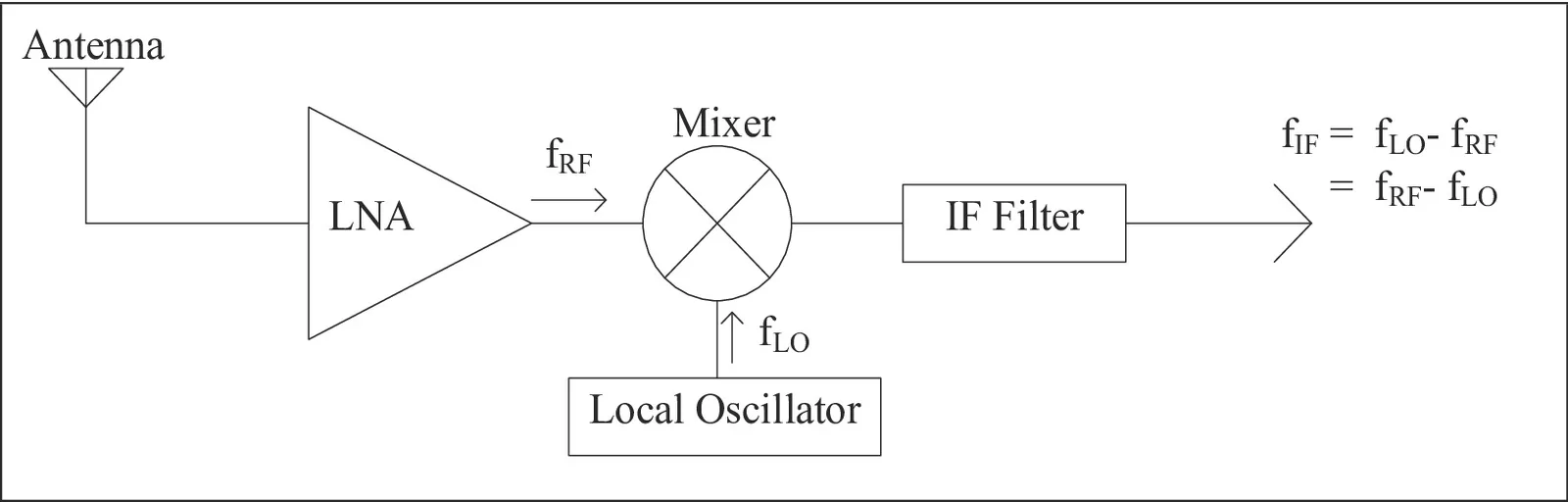
Down conversion system.

Moderate performance and very low power consumption are the primary needs for the systems of short distance wireless communication standards like Zig Bee application in the frequency range of 2.4 GHz. There is a massive effect of scaling in CMOS technology on radio frequency integrated circuits (RFICs), which enhances the operation speed, power consumption and area of IC. So, the integration of base-band digital signal and the RF front end in a single chip depends upon the CMOS process. Among the RF building blocks, mixer is more important than other blocks as a frequency conversion device. The mixer is a 3-port element with 2 input ports and 1 output port for RF, local oscillator (LO) and IF signals respectively as depicted in Fig. 1. Here, the RF signal received from the antenna is applied to the mixer along with the LO signal to obtain the down-converted IF signal at the receiver side.

Gilbert mixers are advantageous for low-power receiver design in application such as electric vehicles (EVs). They provide benefits including high conversion gain, linearity, isolation, which are essential for the wireless communication requirements of EVs, such as infotainment systems, keyless entry, and tire pressure monitoring^1^. Additionally, methods like current reuse and switched biassing can further decrease power consumption and enhance performance.

Gilbert mixer uses in EV include Infotainment systems are used to receive Wi-Fi, satellite, or FM/AM radio transmissions. Keyless Entry Systems: To unlock the car by receiving signals from the key fob. Data from tire pressure sensors is received by tire pressure monitoring systems, or TPMS. V2X communication is used to exchange information with infrastructure and other cars in order to control traffic and ensure safety^2^. Gilbert cell designs are widely used owing to their sensible CG, linearity and NF among the most popular active mixer designs, but with some drawbacks of comparatively high-power supply and high-power consumption owing to the saturation operation of more numbers of stacked transistors. There are many Gilbert mixer design depending upon different technologies and techniques.

The folded technique solves this issue of high supply voltage and high-power consumption by using less stacked transistors, but it results less bandwidth and huge chip area due to matching network inductor^3^. The bulk injection technique^4^ achieves low power supply due to the less current flow through the mixer core. But it gives a high NF and low CG. The bulk pumped mixer^5^ works in low supply voltage due to few stacked transistors uses, in which the CG is poor. The switched biasing technique^6,7^ reduces the noise originating from the tail current transistors. However, it requires a higher power supply due to the increased number of stacked transistors, leading to greater power consumption. The current bleeding technique^8–11^ improves the CG, port to port isolation, noise performance and linearity of the mixer, but with some drawbacks. In ref.^8^, the noise figure as well as power conversion gain are improved, but the chip size is large owing the use of the inductor to resonate out the parasitic capacitance. The high LO to RF isolation and low voltage operation are achieved in ref.^9^, where the NF is not good owing to the replacement of PMOS to NMOS as current bleeding transistors. In^10^, PMOS transistors are employed as a current-bleeding block to improve the conversion gain and noise figure; however, the use of an internal resonant inductor increases the chip area and power consumption. In^11^, the linearity and conversion gain are enhanced, but at the cost of higher power consumption. In^12^, a complementary post-distortion harmonic cancellation technique is adopted to improve the linearity and conversion gain of the mixer, although it results in a high NF. There is an improvement in NF and linearity by using active load and current bleed with source degeneration^13^, but it results low CG and high-power consumption.

In this work, the proposed mixer design is followed by current source helpers and current bleeding technique to solve the conflict in between the input transistor current and load resistor current to achieve high gain, improved linearity and low NF. The design circuit works in a supply voltage of 1.2 V with a power consumption of 0.96 mW in 0.18 μm CMOS technology.

**Fig. 2:**
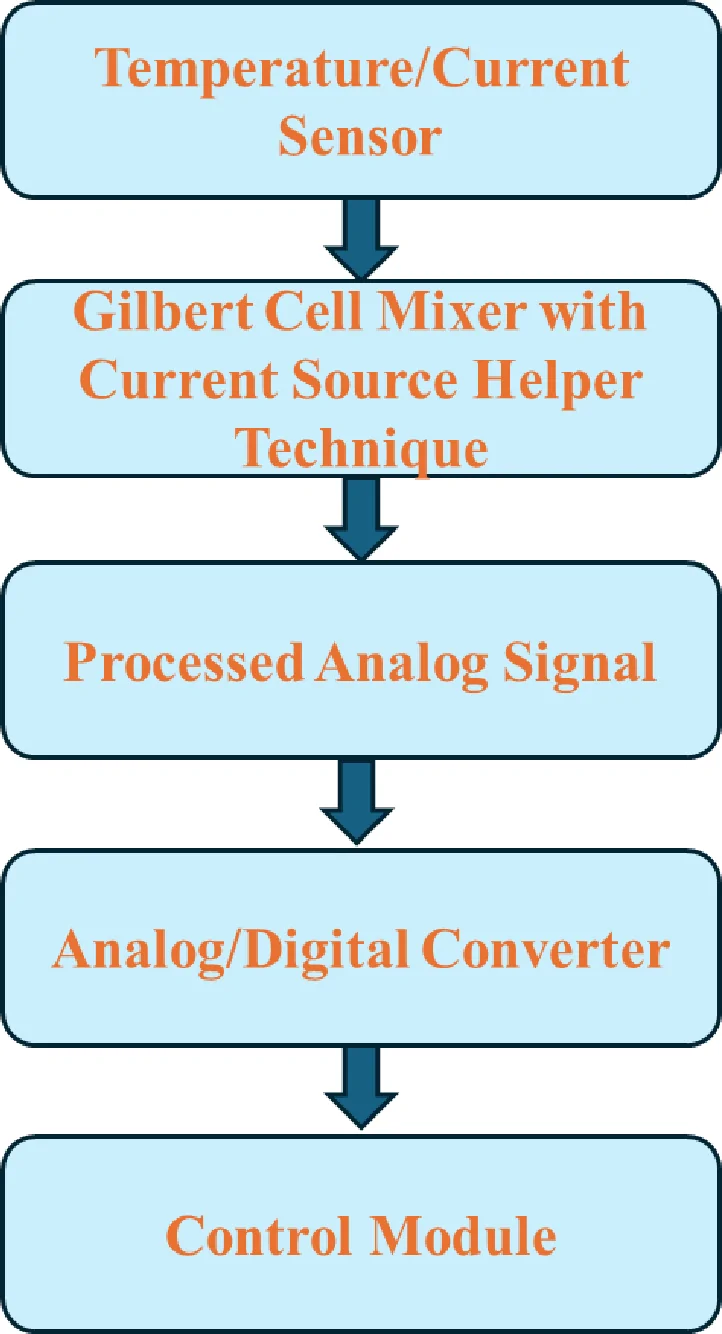
HEV smart sensor module.

This proposed design can be used in sensor fusion in HEV, predictive/adaptive-motor/ fault-tolerant control, wireless sensor networks^14,15^ and edge-analog computation. The smart sensor module in HEV process the analog signal by receiving the data from temperature or current sensor through the Gilbert Mixer with current source helpers. The analog signal is converted into digital signal through A/D converter which is processed by the control module for thermal management, safety measures and motor control which is shown in Fig. 2.

The proposed design with current source helpers and current bleeding technique is explained in section II. The section III signifies the pre-simulation and post-simulation analysis results with the comparison table of diverse CMOS mixer performance and section IV finishes it finally.

## Design and analysis of proposed mixer circuit

### Conventional gilbert cell mixer

**Fig. 3:**
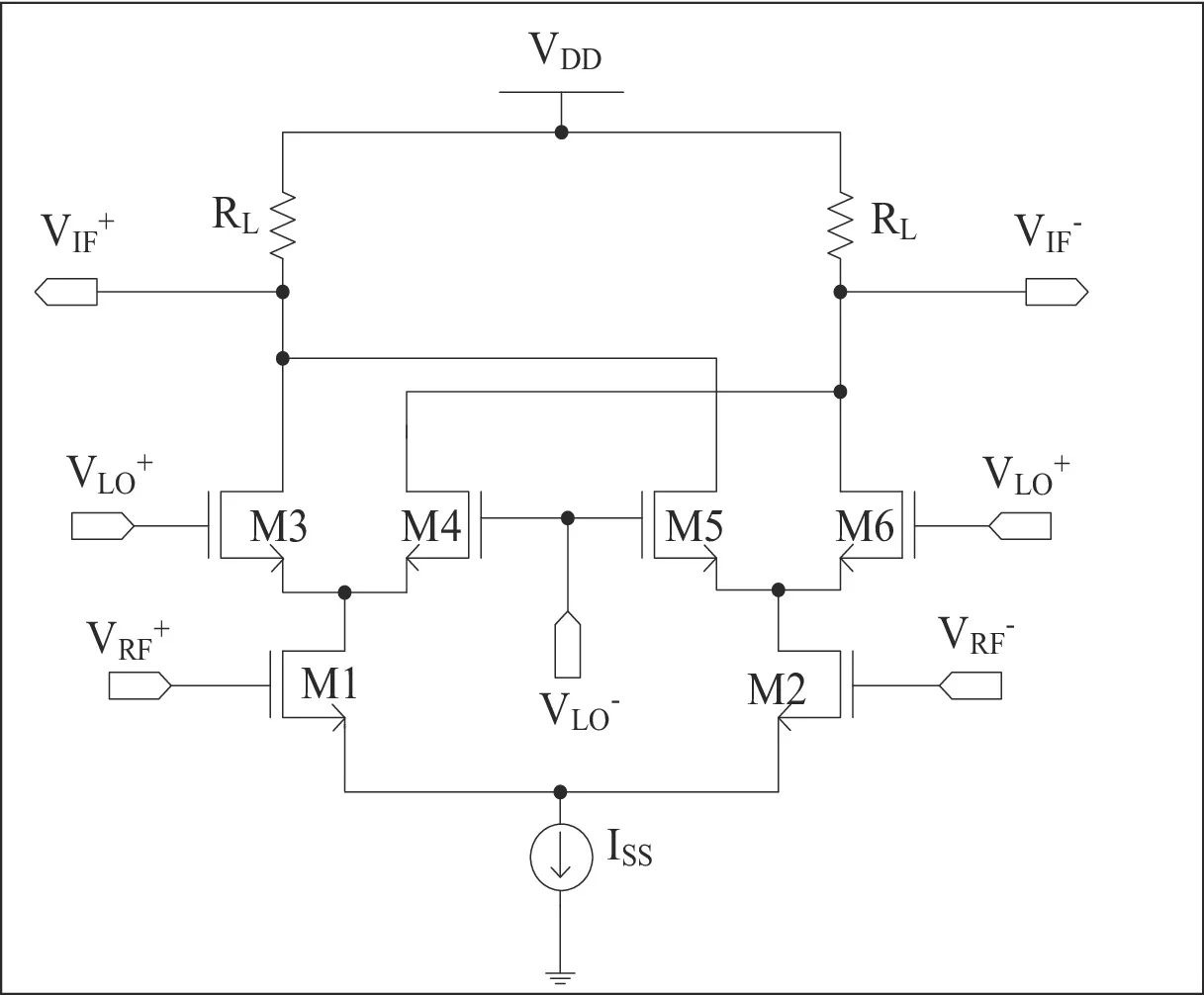
Conventional Gilbert type mixer.

The conventional Gilbert-type mixer is composed of 3 main stages: a transconductance stage, a switching stage, and a load stage, which is represented in Fig. 3. The transconductance stage converts the RF voltage into RF current, which is then modulated by the switching stage to produce an IF current, while the load stage converts the IF current into an IF voltage for efficient signal processing. Here, the IF voltage can be expressed as follows:

1 $$\begin{aligned} \mathop V\nolimits_{{\mathop {IF}\nolimits^{ + } }} & = \left[ {\frac{{\mathop I\nolimits_{{DC}} }}{2} - \mathop g\nolimits_{m} \mathop V\nolimits_{{\mathop {RF}\nolimits^{ + } }} COS\left( {\mathop \omega \nolimits_{{RF}} t} \right)} \right]\mathop F\nolimits_{{square{\text{ }}wave}} \left( t \right)\mathop R\nolimits_{L} \hfill \\ &= {{ }}\frac{{\mathrm{2}}}{\pi }\mathop I\nolimits_{{DC}} \mathop R\nolimits_{L} COS\left( {\mathop \omega \nolimits_{{LO}} t} \right) - \frac{{\mathrm{2}}}{\pi }\mathop g\nolimits_{m} \mathop R\nolimits_{L} \mathop V\nolimits_{{RF^{ + } }} \left\{ {COS\left[ {\left( {\mathop \omega \nolimits_{{RF}} - \mathop \omega \nolimits_{{LO}} } \right)t} \right] + COS\left[ {\left( {\mathop \omega \nolimits_{{RF}} + \mathop \omega \nolimits_{{LO}} } \right)t} \right]} \right\} + ..... \hfill \\ \end{aligned}$$

2 $$\begin{aligned} \mathop V\nolimits_{{\mathop {IF}\nolimits^{ - } }} & = \left[ {\frac{{\mathop I\nolimits_{{DC}} }}{2} - \mathop g\nolimits_{m} \mathop V\nolimits_{{\mathop {RF}\nolimits^{ - } }} COS\left( {\mathop \omega \nolimits_{{RF}} t} \right)} \right]\mathop F\nolimits_{{square{\text{ }}wave}} \left( t \right)\mathop R\nolimits_{L} \hfill \\ &= {{ }}\frac{{\mathrm{2}}}{\pi }\mathop I\nolimits_{{DC}} \mathop R\nolimits_{L} COS\left( {\mathop \omega \nolimits_{{LO}} t} \right) - \frac{{\mathrm{2}}}{\pi }\mathop g\nolimits_{m} \mathop R\nolimits_{L} \mathop V\nolimits_{{RF^{ - } }} \left\{ {COS\left[ {\left( {\mathop \omega \nolimits_{{RF}} - \mathop \omega \nolimits_{{LO}} } \right)t} \right] + COS\left[ {\left( {\mathop \omega \nolimits_{{RF}} + \mathop \omega \nolimits_{{LO}} } \right)t} \right]} \right\} + ..... \hfill \\ \end{aligned}$$

Referring to the Fig. 3, consider

3 $$\mathop V\nolimits_{{\mathop {RF}\nolimits^{ + } }} = \frac{{\mathop V\nolimits_{{RF}} }}{2}{\text{ and }}\mathop V\nolimits_{{\mathop {RF}\nolimits^{ - } }} = - \frac{{\mathop V\nolimits_{{RF}} }}{2}$$

The IF output voltage is represented as:

4 $$\mathop V\nolimits_{{IF}} = \mathop V\nolimits_{{\mathop {IF}\nolimits^{ + } }} - \mathop V\nolimits_{{\mathop {IF}\nolimits^{ - } }}$$

Applying (1), (2) and (3) in (4), VIF is represented as

5 $$\mathop V\nolimits_{{IF}} = - \frac{{\mathrm{2}}}{\pi }\mathop g\nolimits_{m} \mathop R\nolimits_{L} \mathop V\nolimits_{{RF}} \left\{ {COS\left[ {\left( {\mathop \omega \nolimits_{{RF}} - \mathop \omega \nolimits_{{LO}} } \right)t} \right] + COS\left[ {\left( {\mathop \omega \nolimits_{{RF}} + \mathop \omega \nolimits_{{LO}} } \right)t} \right]} \right\} + .....$$

So, voltage CG will be

6 $$C.G{\text{ = }}\frac{2}{\pi }\mathop g\nolimits_{m} \mathop R\nolimits_{L}$$

where *I*_*DC*_ is the dc current, *g*_*m*_ is the transconductance of RF stage, *F*_*square wave*_ is the square wave signal function applied through the LO input and *R*_*L*_ is the load resistor.

Designing a Gilbert mixer is challenging because linearity and noise requirements demand high input transistor current, whereas achieving high gain requires low load resistor current to allow larger resistor values. Current source helpers are therefore employed to balance these conflicting design requirements. As well as the current bleeding concept is implemented for the improvement of CG and noise performance.

### Proposed gilbert mixer

**Fig. 4:**
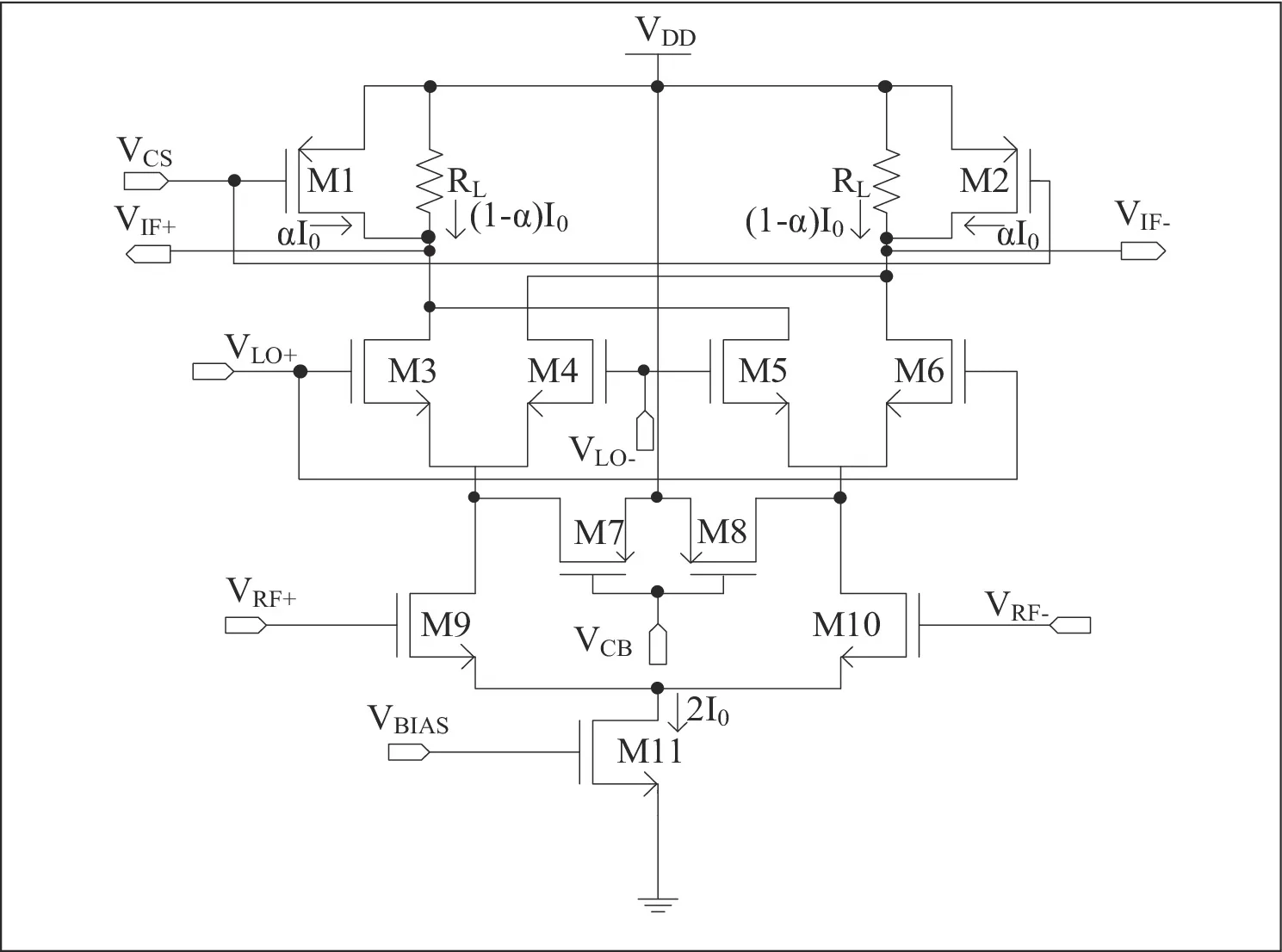
Proposed mixer design.

The proposed mixer is implemented by the current source helpers and current bleeding technique, which is shown in Fig. 4. This design includes a load stage (R_L_) in parallel with current source helpers (M1–M2), switching stage (LO) formed by transistors (M3–M6), a current bleeding stage (M7-M8), a transconductance stage (RF) using M9-M10 and tail current source stage (M11). Current bleeding techniques and helper current sources significantly enhance the mixer performance in terms of gain, noise figure, and linearity.

### Current source helpers

The load resistors (R_L_) are in parallel connection through an extra added current source helpers (M1-M2) to get large resistor values. So, it results a high CG for the mixer design as per in (6). In the Fig. 4, if the tail current source transistor (M11) current is ID_11_ (= 2I_0_) and each transistor M1 and M2 transmits a fraction of current αI0, then the load resistors (R_L_) will have a large value^16^. This R_L_ is expressed in (7), which results an increased gain with the increment value of α.

7 $$\mathop R\nolimits_{L} = \frac{{\mathop V\nolimits_{0} }}{{\left[ {\left( {1 - \alpha } \right)\mathop I\nolimits_{0} } \right]}}$$

where *V*_*0*_ is the extreme acceptable drop crosswise *R*_*L*_. The larger value of *R*_*L*_ also diminishes the involvement of its input mentioned noise, which is expressed in (8).

8 $$\overline{{\mathop V\nolimits_{{n,in}}^{2} }} {\text{ }} = {\text{ }}\mathop \pi \nolimits^{2} {\text{ }}kT\left( {\frac{\gamma }{{\mathop g\nolimits_{{m9 - 10}} }} + \frac{2}{{\mathop g\nolimits_{{m9 - 10}}^{2} \mathop R\nolimits_{L} }}} \right)$$

where *k* is the Boltzmann constant, *T* is temperature in Kelvin, γ is the excess noise coefficient, *g*_*m9-10*_ is the transconductance of M9 and M10 transistors and *R*_*L*_ is the load resistor.

The output node noise voltage resulting from the current source helper (M1-M2) is also taken into account (9).

9 $$\overline{{\mathop V\nolimits_{{n,out}}^{2} }} {\text{ }} = {\text{ }}4kT\gamma \frac{{2\alpha \mathop I\nolimits_{0} }}{{\mathop V\nolimits_{0} }}\mathop R\nolimits_{L}^{2} + 4kTR_{L}$$

As the load resistor *R*_*L*_ is directly proportional to the voltage CG, the noise power in (9) should be regularized to *R*^*2*^_*L*_, as shown in (10).

10 $$\begin{aligned} \frac{{\overline{{\mathop V\nolimits_{{n,out}}^{2} }} }}{{\mathop R\nolimits_{L}^{2} }}&= {\text{ }}4kT\gamma \frac{{2\alpha \mathop I\nolimits_{0} }}{{\mathop V\nolimits_{0} }} + \frac{{4kT}}{{\mathop R\nolimits_{L} }} \hfill \\ &= {\text{ }}4kT\frac{{\mathop I\nolimits_{0} }}{{\mathop V\nolimits_{0} }}\left( {2\alpha \gamma + 1 - \alpha } \right) \hfill \\ &= {\text{ }}4kT\frac{{\mathop I\nolimits_{0} }}{{\mathop V\nolimits_{0} }}\left[ {\left( {2\gamma - 1} \right)\alpha + 1} \right] \hfill \\ \end{aligned}$$

As α increases, the noise from helpers (M1–M2) and associated resistors also increases. Therefore, the current sources operate near saturation to reduce transconductance and noise current, improving the proposed circuit’s linearity.

### Current bleeding technique

The current bleeding transistors (M7–M8) are added to the transconductance stage to improve the conversion gain by increasing the RF current. This RF current is used to operate the transconductance transistors (M9–M10) in the saturation region to preserve the total current of the mixer, while there is a reduction of LO current. The PMOS transistors M7 and M8 proceed as the paths of current bleedings to create more current flow into the transistors M9 and M10. Consequently, the CG increases and also there is noise reduction owing to the low noise of PMOS transistors over NMOS transistors.

But, the proper size of transistors in the current bleeding stage and LO switching stage (M3–M6) is chosen to reduce the parasitic effects at the source of switching stage. The overdrive voltage diminishes due to the reduction of current in switching stage^17–20^. Thus, the faster operation of LO switches to make the flicker noise (1/f) small. The conversion gain of the mixer design is expressed in (11).

11 $$C.G = {\text{ }}\frac{{\mathrm{2}}}{\pi }\left( {\mathop g\nolimits_{{m,n}} + \mathop g\nolimits_{{m,p}} } \right)\mathop R\nolimits_{L}$$

where *g*_*m, n*_, *g*_*m, p*_ and *R*_*L*_ denote transconductance of NMOS transistors (M9–M10) of RF stage and PMOS transistors (M7–M8) of current bleeding stage and load resistance respectively. This current bleeding path progresses current distribution between the RF and switching stages, thereby reducing compression and higher-order distortion in the dominant nonlinear paths. The IIP3 enhancement is attained through the combined current source helper biasing, current bleeding based current redistribution, boosted RF transconductance linearity, and reduced nonlinearity in the switching stage.

## Proposed design result analysis

The circuit design presented in this work is executed using UMC 0.18 μm technology with the Cadence design tool. The device parameters are displayed in Table 1 for this proposed work. The parameters such as CG, IIP3 and NF of the proposed design are simulated in 2.4 GHz RF frequency with a LO power of 5 dBm.

The post-layout simulations are performed after extracting the layout parasitic (RCX), including parasitic resistance, capacitance, inductance followed by DRC, LVS check of the layout design by using the parasitic extraction tool Assura in Cadence. The extraction tool generates an extracted netlist (av_extracted) containing the original circuit elements along with the parasitic. This extracted netlist is used for post-layout simulations (config_view) to evaluate the actual performance of the mixer under realistic layout effects.

**Table 1:** Device parameters of the mixer design.

Device parameters	Proper value
(W/L) _M1, M2_	50/0.18 μm
(W/L) _M3 – M6_	25/0.18 μm
(W/L) _M7, M8_	40/0.18 μm
(W/L) _M9 – M11_	25/0.18 μm
RL	1 KΩ

**Fig. 5:**
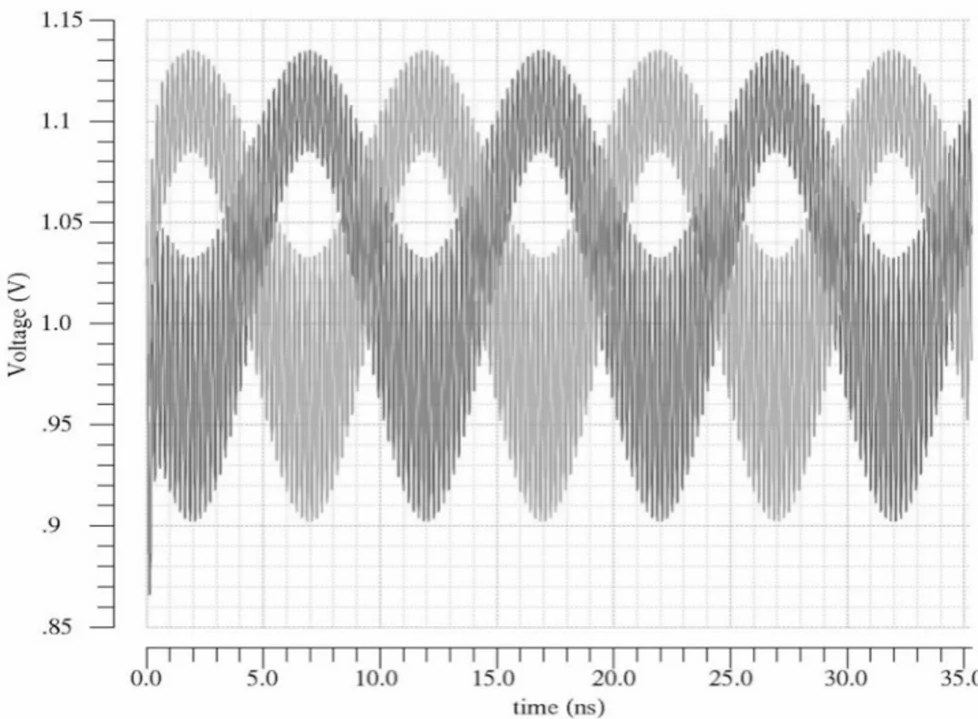
Transient analysis of proposed design.

Figure 5 shows the transient response of the proposed design by using Transient analysis, which represents the IF frequency of 100 MHz. So, it is verified that the design works at RF of 2.4 GHz with 2.3 GHz LO frequency. The plot contains a noticeable high-frequency component due to the residual RF and LO feedthrough as well as higher-order mixing products inherent to the mixer operation.

**Fig. 6:**
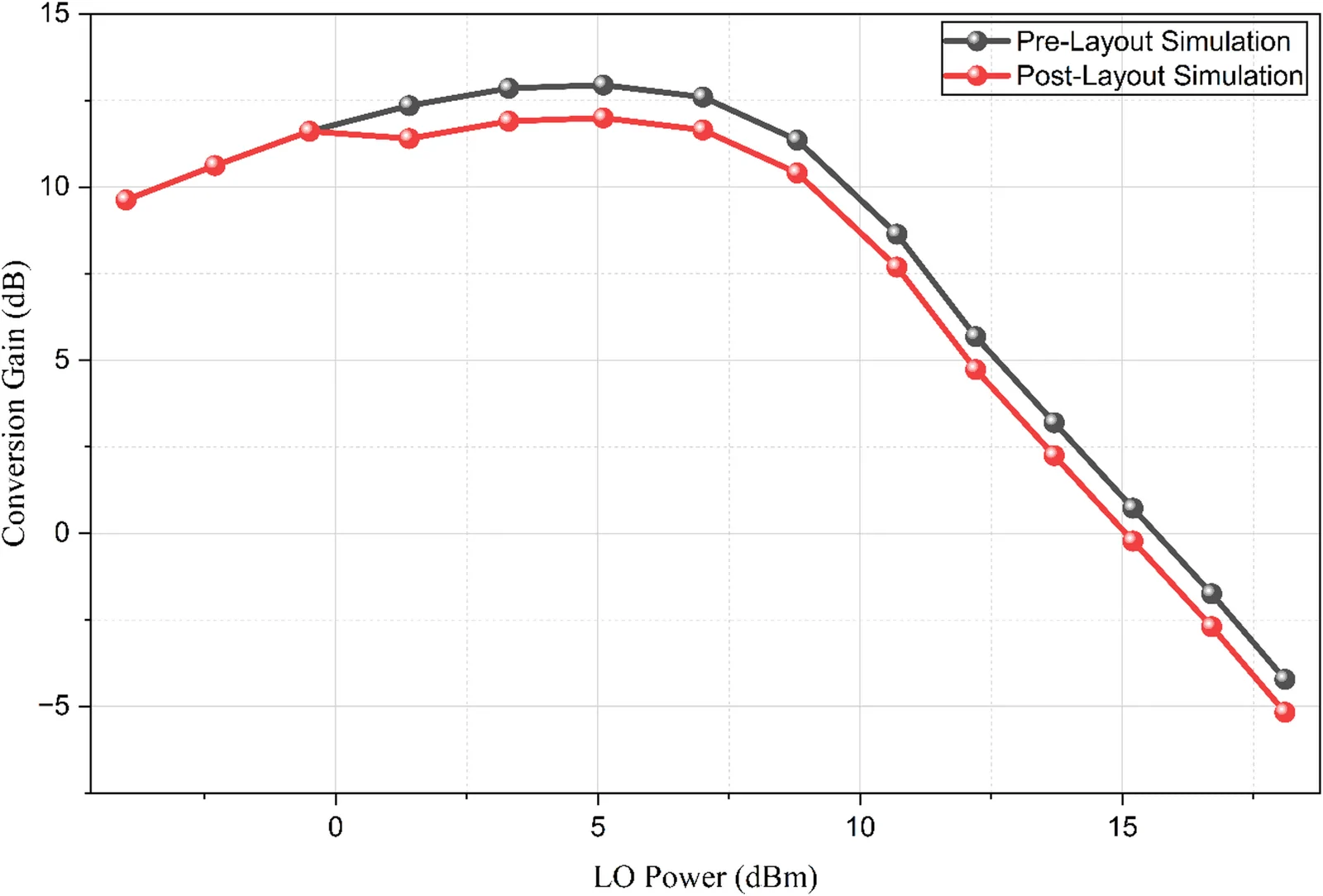
CG versus LO power (plo) of proposed design.

The Fig. 6 illustrates the CG as a function of LO power. The graph represents a peak CG of 12.95 dB and 12 dB achieved at an LO power level of 5 dBm by the help of periodic steady state (PSS) and periodic AC (PAC) analysis in pre-layout and post-layout simulations respectively.

**Fig. 7:**
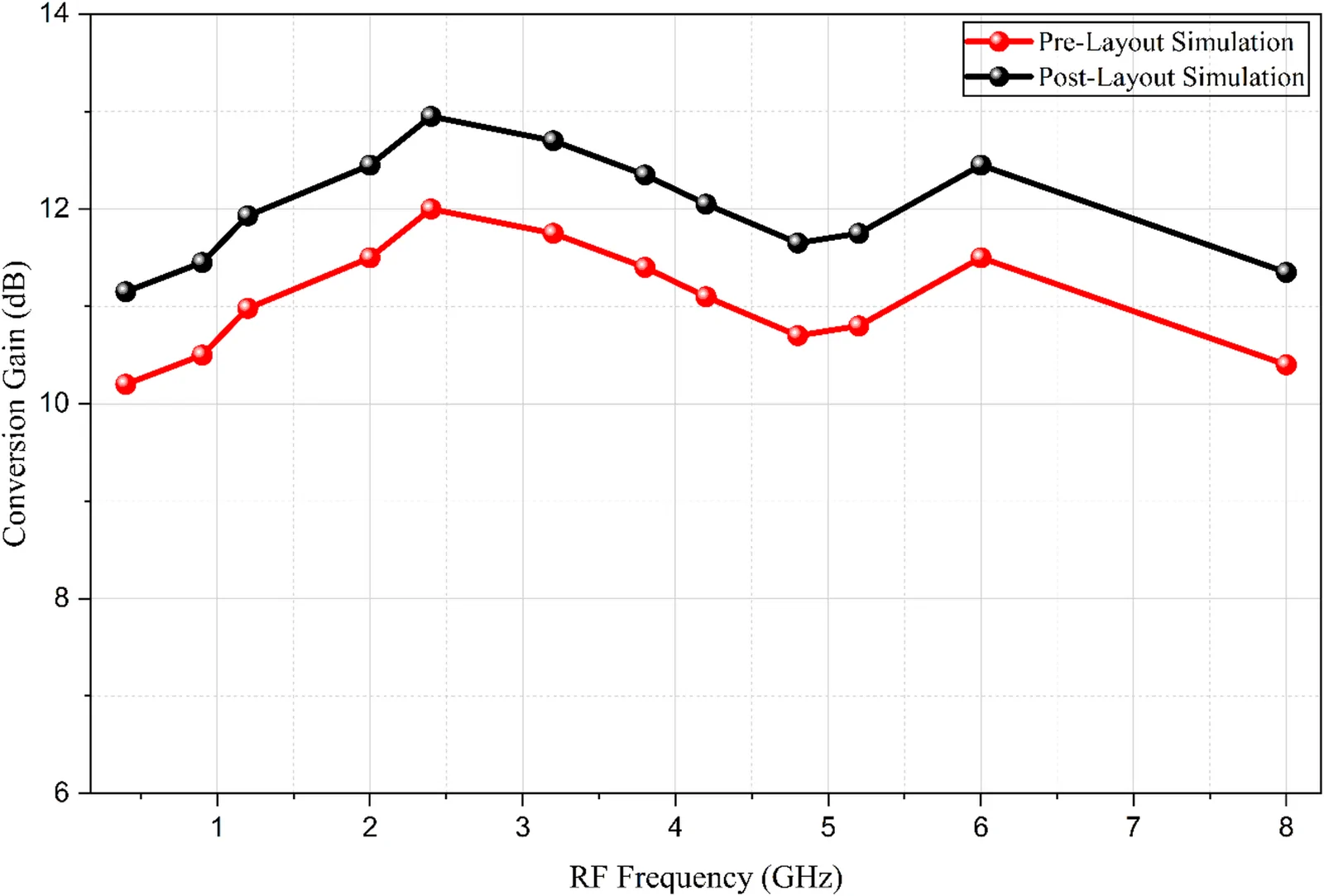
CG versus RF frequency of proposed design.

The Fig. 7 demonstrates the CG as a function of RF frequency sweep from 0.4 GHz to 8 GHz for the applications of HEV (V2X, Bluetooth, Wi-Fi) and wireless sensor networks (Zigbee, LoRa). The graph signifies a peak CG of 12.95 dB and 12 dB at RF frequency of 2.4 GHz in pre-layout and post-layout simulations respectively.

The wideband response of the designed mixer is mainly achieved through the use of a wideband Gilbert mixer architecture, optimized transistor sizing, and proper impedance matching at the RF and LO ports. The absence of narrowband resonant components such as inductive LC tanks also helps keep stable conversion gain over an extensive frequency range. Furthermore, careful biasing and layout optimization reduce parasitic effects, enabling the mixer to operate efficiently from 0.4 GHz to 8 GHz.

**Fig. 8:**
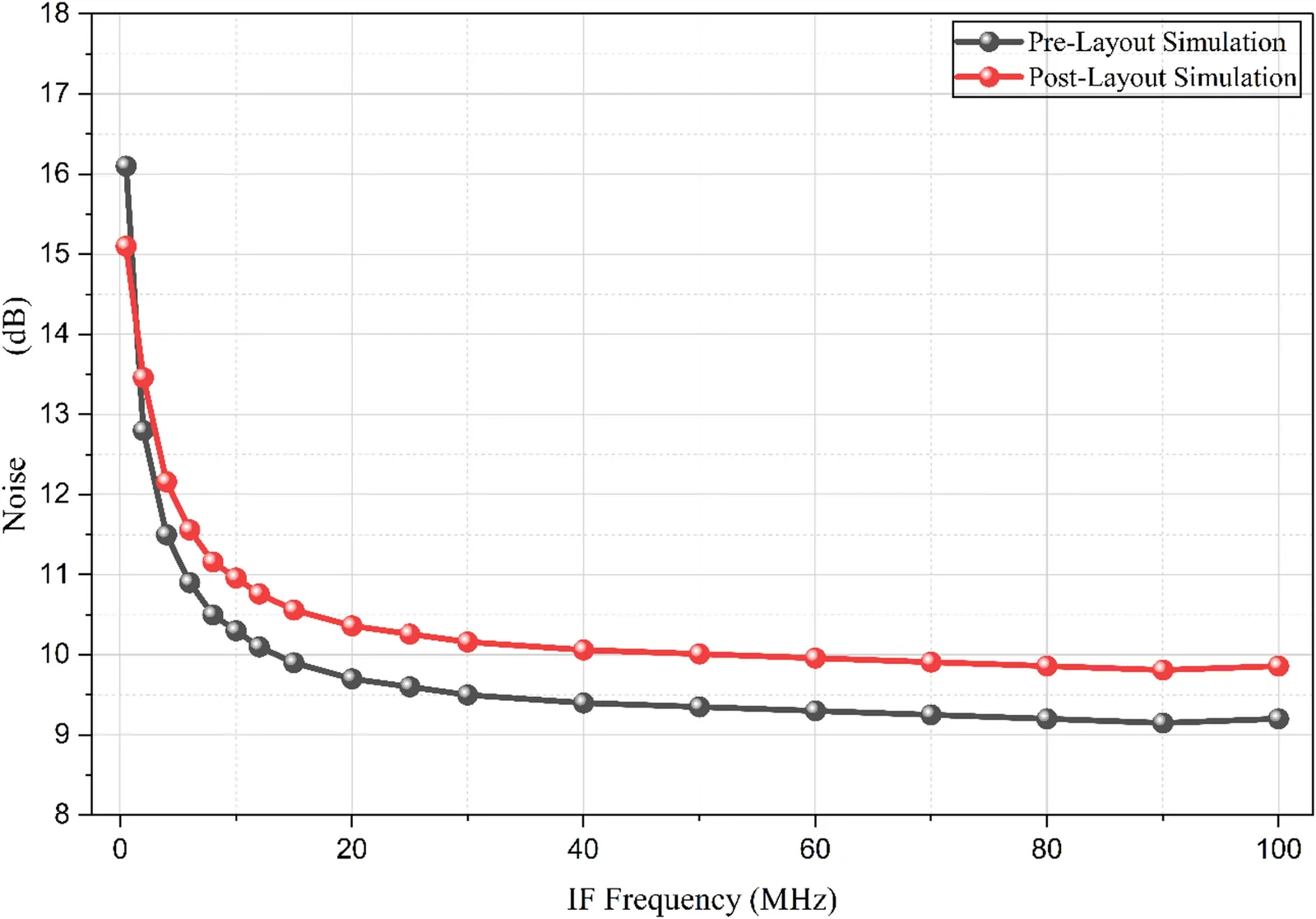
Noise figure vs. IF frequency of planned mixer.

Figure 8 displays the NF plotted against IF frequency indicating a NF of approximately 9.2 dB and 9.86 dB at an IF frequency of 100 MHz in pre-layout and post-layout simulations respectively.

**Fig. 9:**
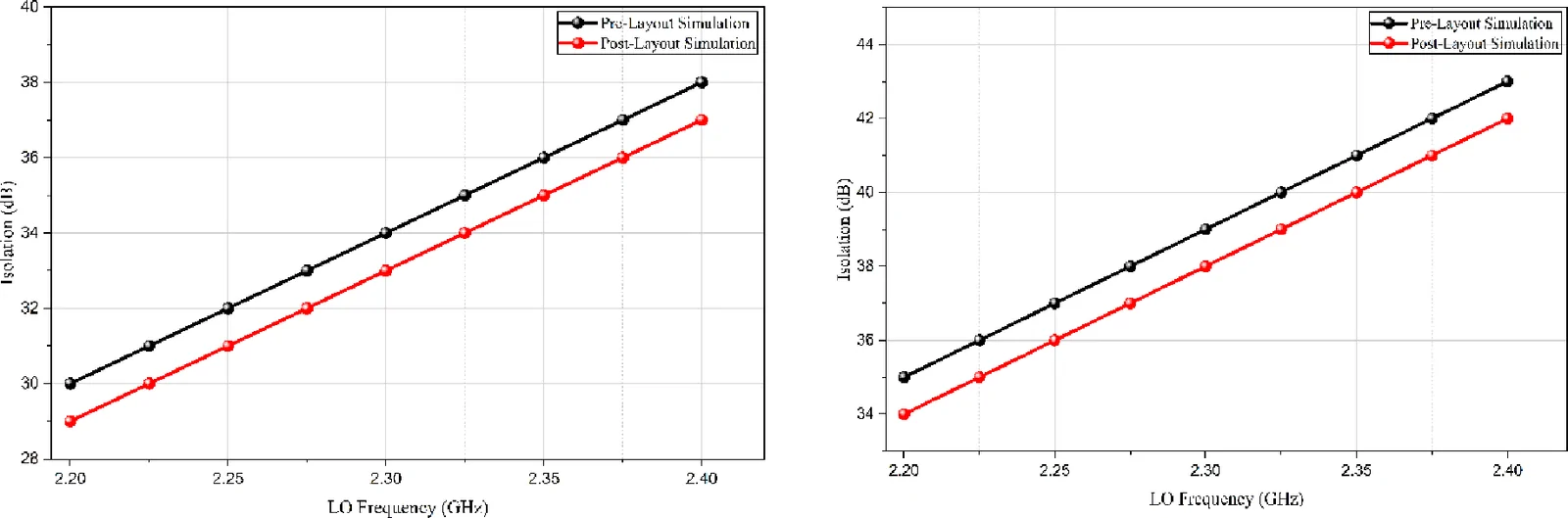
(**a**) LO to IF isolation. (**b**) LO to RF isolation.

The proposed design achieves LO–IF isolation of 29–37 dB and LO–RF isolation of 34–42 dB within the desired frequency band, as shown in Fig. 9a and b, respectively. The figure also presents both pre-layout and post-layout simulation results for comprehensive evaluation of LO feedthrough suppression.

**Fig. 10:**
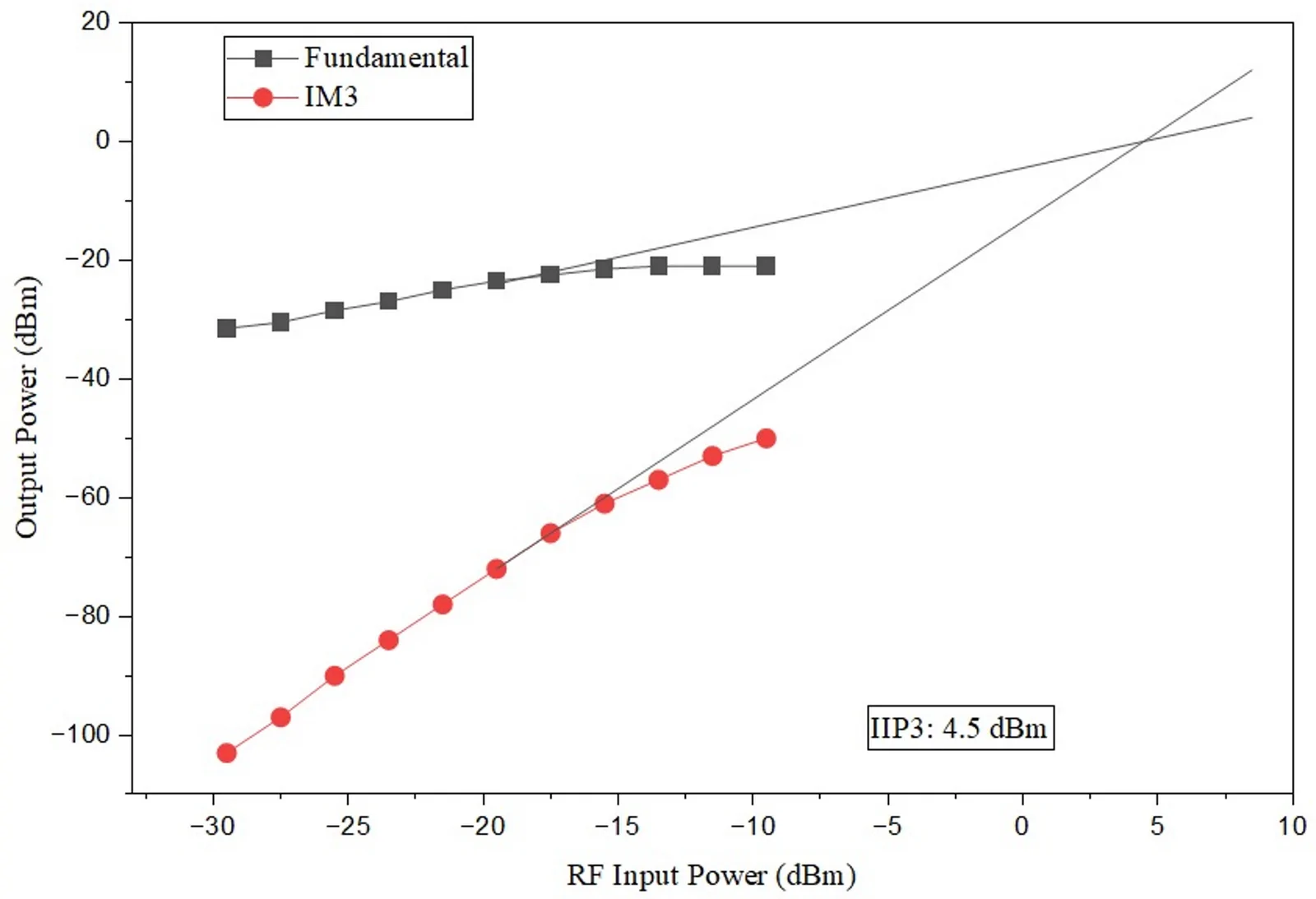
IIP3 of projected mixer.

**Fig. 11:**
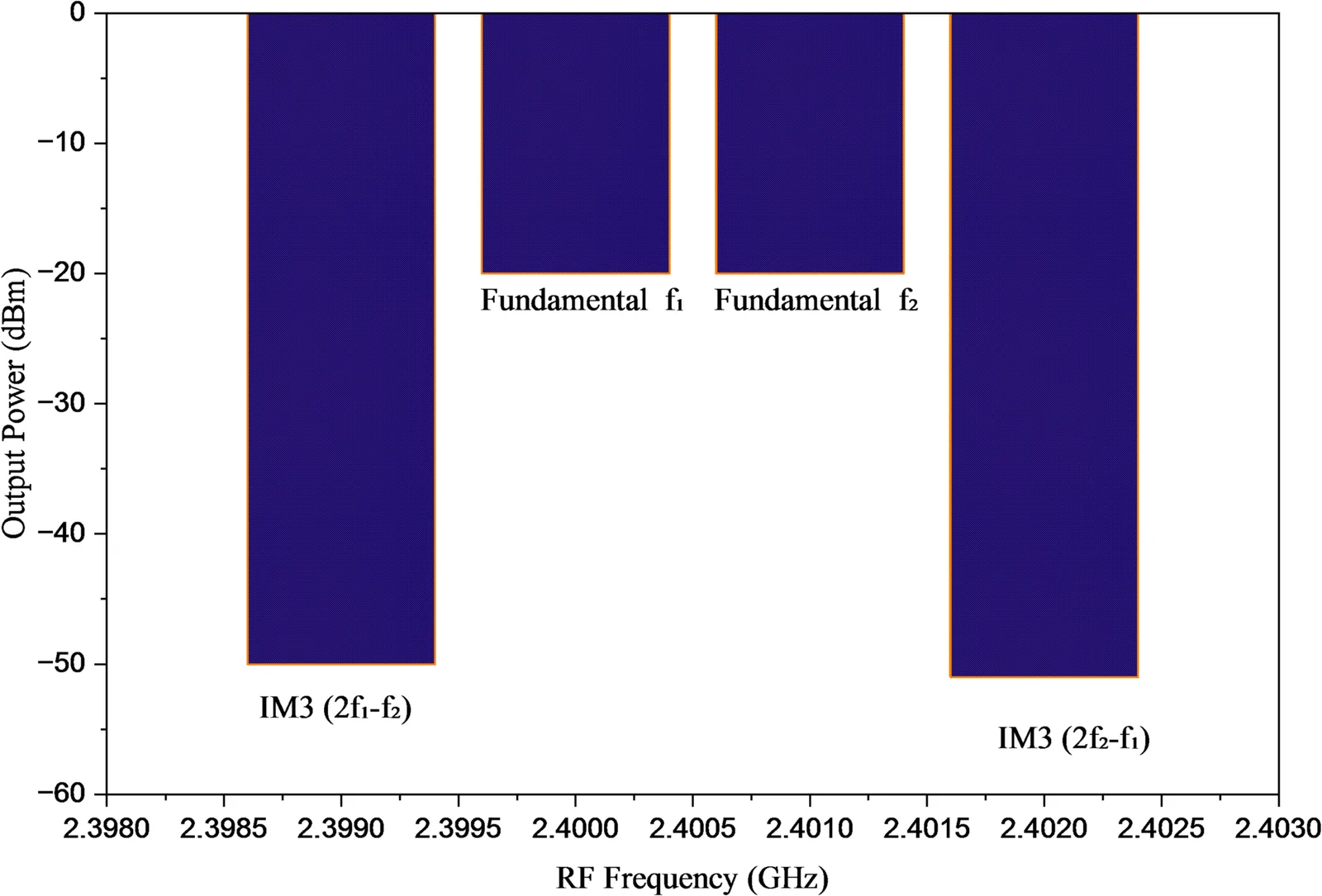
Two-tone test spectrum plot of projected mixer.

**Fig. 12:**
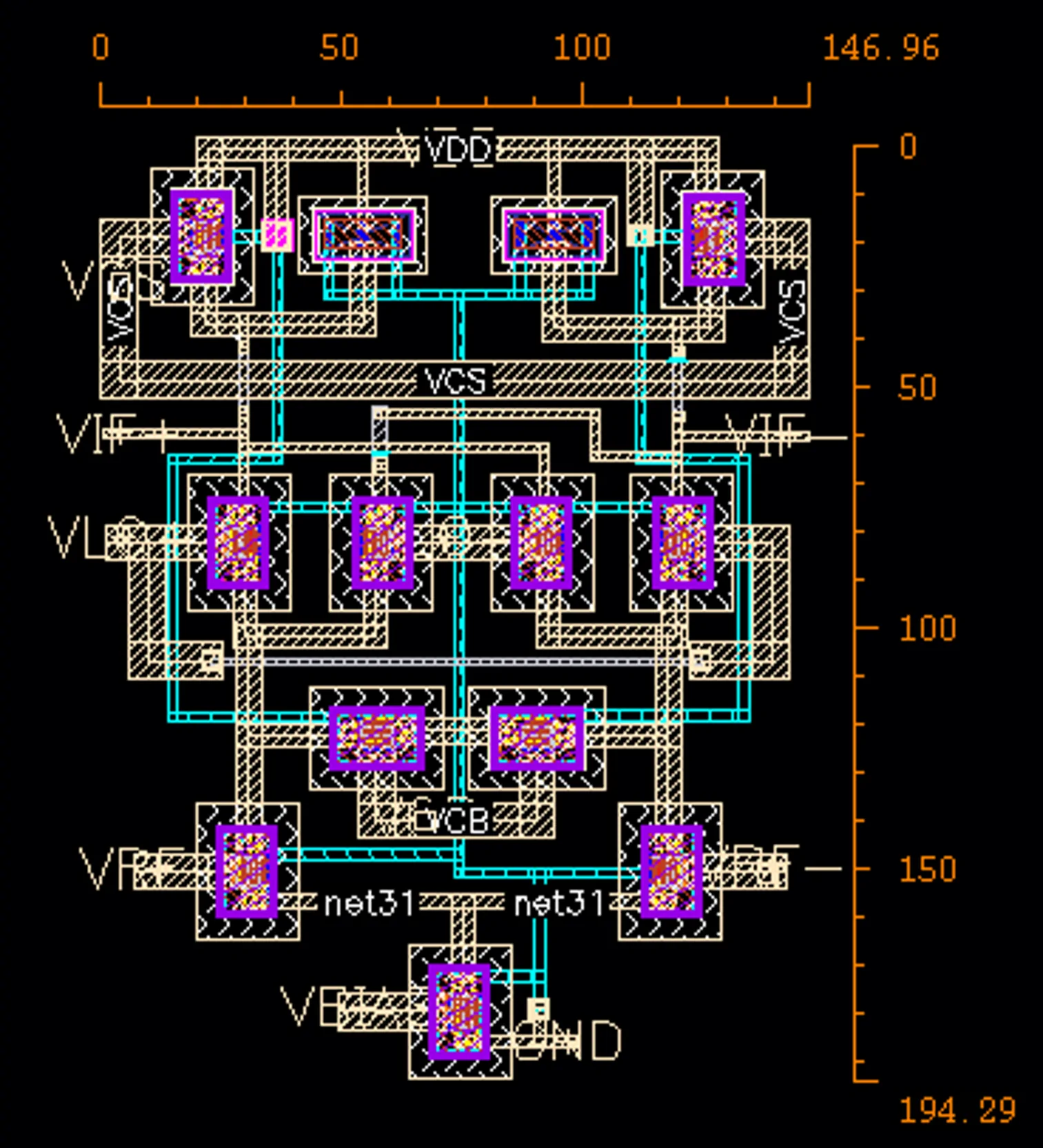
Layout diagram of proposed design.

Figure 10 presents the IIP3 plot (RF power vs. IF power) of the mixer design demonstrating an IIP3 value of 4.5 dBm by using Quasi PSS and Quasi PAC analysis. This indicates that at an input RF power of 4.5 dBm, the 3rd order intermodulation products theoretically become equal to the fundamental output signal, thereby defining the mixer’s linearity performance. The two-tone spectrum plot of the proposed mixer, shown in Fig. 11, represents the fundamental and 3rd order intermodulation components and is used to visually validate the mixer’s linearity as well as the reported IIP3 value. The layout diagram of the proposed mixer is shown in Fig. 12, occupying an area of 0.15 × 0.19 mm^2^.

An ardent comparison between pre-layout and post-layout simulation results is tabulated in Table 2 for key performance parameters of the proposed mixer.

**Table 2:** Proposed design pre-layout and post-layout simulation results.

Design parameters	Pre-layout simulation	Post-layout simulation
Conversion gain (dB)	12.95	12
Noise figure (dB)	9.2	9.86
IIP3 (dBm)	5	4.5
Power consumption (mW)	0.85	0.96
LO-to-IF (dB)	30–38	29–37
LO-to-RF (dB)	35–43	34–42

The simulation results of the proposed mixer under different process corners and temperature variations are summarized in Table 3. The results indicate only minor variations in the mixer performance parameters, demonstrating the robustness of both proposed techniques.

From the implementation results, it is found that the planned design consumes 0.96 mW power from a 1.2 V power supply. It is seen that the mixer gives the CG of 12 dB, while the NF and IIP3 are 9.86 dB and 4.5 dBm respectively.

**Table 3:** Proposed design post-simulation in three process corners with temperature variations.

Process corner Design parameters	Typical typical (TT) at 27 °C	Fast fast (FF) at – 40 °C	Slow slow (SS) at 85 °C
Conversion gain (dB)	12	10.1	12.4
Third order input intercept point (dBm)	4.5	5.05	4.25
Noise figure (dB)	9.86	9.1	10.5
Supply voltage (V)	1.2	1.2	1.2
Power consumption (mW)	0.96	1.5	0.91

The performance comparison of various CMOS mixers is summarized in Table 4. In contrast to other mixers listed in Table 4, this mixer design achieves enhanced linearity, higher CG and low NF by using the current source helpers and current bleeding technique.

**Table 4:** Performance summary of CMOS mixers.

Refs.	CMOS process	Frequency (GHz)	Conversion gain (dB)	DSB NF (dB)	Supply voltage (V)	IIP3 (dBm)	Power (mW)
^3^_M_	0.18 μm	0.2–16	5.3	-	1.8	-	15
^6^_M_	0.13 μm	2.4	7.6	10.9	1.8	-5	8.1
^8^_M_	0.18 μm	24	8.376	11.6	1.8	-8.4	5.65
^9^_M_	0.13 μm	2.4	7.5	15	0.45	1	0.572
^10^_M_	0.18 μm	3.16–3.69	7.5–10.1	8.8–12.5	1.8	-6.2	13.24
^11^_M_	0.25 μm	0.9–10.6	7.6–9.6	13.1–13.8	1	-4	18.3
^12^_S_	0.18 μm	2.4	14.36	12.57	1.2	5.05	1.25
^13^_S_	0.13 μm	5.825	10.26	9.95	1.3	3.08	2.5
**[This work]s**	**0.18 μm**	**2.4**	**12**	**9.86**	**1.2**	**4.5**	**0.96**

*M: Measured data; S: Simulated data.

## Conclusion

This paper discusses the proposed work to address the critical requirement of receivers to detect weak signals. Reception issues and interference can lower the EV’s wireless communication systems’ overall usability. Current receiver technologies are one way to address this issue, as they reduce interference and improve signal reception. The current source helpers are used with the parallel connection of the load resistors to get large resistor values for the improvement in conversion gain of the mixer. The current bleeding technique is also used with the RF stage to enhance the CG, linearity and noise performance of the design. The proposed design results a power consumption of 0.96 mW from a power supply of 1.2 V with a CG of 12 dB, NF of 9.86 dB and IIP3 of 4.5 dBm a with good isolation from LO to IF of (29–37) dB and from LO to RF of (34–42) dB. Therefore, the proposed mixer offering enhanced gain, linearity, and noise performance, is well-matched as a key component for low voltage, low power receiver designs in applications such as electric vehicles (EVs) and sensor networks. One limitation of this work is its larger area occupancy due to the use of a larger number of transistors. Furthermore, the result analysis has been supported only at a particular frequency of 2.4 GHz rather than over a wider frequency range, which restricts the mixer’s performance across diverse frequencies.

## Data Availability

The datasets used and/or analyzed during the current study available from the corresponding author on reasonable request.

## References

[1] Kurkin, A. et al. Battery management system for electric vehicles: comprehensive review of circuitry configuration and algorithms. *World Electr. Veh. J.* **16**, 1–34 (2025).

[2] Deng, Z. et al. Design of a 60-kW EV dynamic wireless power transfer system with dual transmitters and dual receivers. *IEEE J. Emerg. Sel. Top. Power Electron.* **12**, 316–327 (2023).

[3] Chang, F. C., Huang, P. C., Chao, S. F. & Wang, H. A low power folded mixer for UWB system applications in 0.18 µm CMOS technology. *IEEE Microw. Wirel. Compon. Lett.* **17**, 367–369 (2007).

[4] Liang, K. H., Chan, Y. J. & Chang, H. Y. A 0.5–7.5 GHz ultra-low-voltage, low-power mixer using bulk injection method by 0.18 µm CMOS technology. *IEEE Microw. Wirel. Compon. Lett.* **17**, 531–533 (2007).

[5] Kuo, C. L., Huang, B. J., Kuo, C. C., Lin, K. Y. & Wang, H. A 10–35 GHz low power bulk driven mixer using 0.13 µm CMOS process. *IEEE Microw. Wire-less Compon. Lett.* **18**, 455–457 (2008).

[6] Kim, J. H., An, H. W. & Yun, T. Y. A low-noise WLAN mixer using switched biasing technique. *IEEE Microw. Wirel. Compon. Lett.* **19**, 650–652 (2009).

[7] Rout, S. S., Mohapatra, S. K. & Sethi, K. Design of cascode mixer based on bulk injection and switched biasing techniques in 180 nm CMOS process for a high-performance receiver front end. *J. Low Power Electron.* **14**, 49–56 (2018).

[8] Chang, Y. H., Huang, C. Y. & Chiang, Y. C. A 24 GHz down-conversion mixer with low noise and high gain, IEEE Conferences on Microwave Integrated Circuits, Netherlands, (2012).

[9] Tan, G. H., Sidek, R. M., Ramiah, H., Chong, W. K. & Lioe, D. X. Ultra-low-voltage CMOS-based current bleeding mixer with high LO-RF isolation. *Sci. World J.* **2024**, 1–5 (2014). 10.1155/2014/163414 PMC4150493 25197694

[10] Jung, G. Y., Shin, J. H. & Yun, T. Y. A low-noise UWB CMOS mixer using current bleeding and resonant inductor. *Microw. Opt. Technol. Lett.* **49**, 1595–1597 (2007).

[11] Yeh, C. I., Feng, W. S. & Hsu, C. Y. 0.9–10.6 GHz UWB mixer using current bleeding for multi-band application. *Electron. Lett.* **50**, 186–187 (2014).

[12] Gladson, S. C. & Bhaskar, M. A low-power RF mixer with harmonic cancellation for IEEE 802.15.4 portable, wearable wireless applications. *AEU-International J. Electron. Commun.* **124**, 153335 (2020).

[13] Satyanarayana, R. V. S. & Avvaru, S. Design and optimization of double balanced gilbert cell mixer in 130 nm CMOS process. *Solid State Electron. Lett.* **2**, 129–139 (2020).

[14] Mishra, A., Sahoo, U. K. & Maiti, S. Robust structured sparsity based fused lasso estimator with sensor node position uncertainty. *IEEE Trans. Circuits Syst. II Express Briefs*. **71**, 2449–2453 (2024).

[15] Mishra, A., Sahoo, U. K. & Maiti, S. Sparsity-enabled radio tomographic imaging using quantized received signal strength observations. *Digit. Signal Proc.* **127**, 103576 (2022).

[16] Rout, S. S. & Sethi, K. DTMOS based Gilbert mixer design for MICS receiver using current source helpers and switched biasing technique. *Sadhana* **45**, 1–7 (2020).

[17] Sravanthi, P. & Chandra, A. Current-bleeding folded Gilbert RF mixer design for wireless applications. in *Proceedings of IEEE Conference on Information and Communication Technologies*, pp. 1044–1048 (2013).

[18] Rout, S. S. & Sethi, K. A high gain, low power and low noise down conversion mixer using 0.18 µm CMOS process. *Modell Measure Control Ser. Gen. Phys. Electr. Appl.* **90**, 353–367 (2017).

[19] Darabi, H. & Chiu, J. A noise Cancellation technique in active RF CMOS Mixers. *IEEE J. Solid-State Circuits*. **40**, 2628–2632 (2005).

[20] Rout, S. S., Mohapatra, S. K. & Sethi, K. Design of 2.4 GHz improved current reuse gilbert mixer with source degeneration technique. *Wireless Pers. Commun.* **122**, 3875–3887 (2022).

